# Squamous cell carcinoma implantation in gastrostomy orifice. Case report

**DOI:** 10.31744/einstein_journal/2020RC5409

**Published:** 2020-10-29

**Authors:** Ana Flávia Azevedo Querichelli, Fernanda Luiza Faria, Laura Ferreira Martinez, Eumildo de Campos

**Affiliations:** 1 Faculdade de Medicina de São José do Rio Preto São José do Rio PretoSP Brazil Faculdade de Medicina de São José do Rio Preto, São José do Rio Preto, SP, Brazil.

**Keywords:** Gastrostomy, Carcinoma, squamous cell, Neoplasm metastasis, Head and neck neoplasms, Enteral nutrition

## Abstract

Percutaneous endoscopic gastrostomy is used to provide enteral nutritional support for patients with obstructive oropharyngeal or esophageal neoplasms. The placement of the catheter is considered safe, with few complications. Despite this, a specific complication that is considered rare, has been increasingly described in the literature, *i.e*., metastasis of head and neck cancer in the gastrostomy stoma. In this report, we described a case of metastasis of squamous cell carcinoma of the larynx in the gastrostomy site, and discussed the possible etiologies and alternatives, seeking to reduce the incidence of this complication.

## INTRODUCTION

Endoscopic percutaneous gastrostomy (EPG) is used as an alternative enteral feeding route when the oral route is damaged, and the patient is unable to adequately nourish themselves, seeking to avoid malnutrition and cachexia.^(^^1^^)^ It is an alternative to surgical gastrostomy or nasogastric tube in patients who require long-term nutritional support.^(^^1^^)^ It is indicated in cased of obstructive neoplasms of the larynx, pharynx, or esophagus, swallowing difficulties due to neurological disease or radiation therapy, and facial trauma.^(^^2^^)^

When the probe used to perform endoscopic gastrostomy is manipulated in the oropharyngeal region with a neoplastic lesion, it may come into contact with the tumor, and induce metastases by direct implantation of neoplastic cells in a site opened by incision on the gastric surface when passing the probe,^(^^3^^)^ or due to scaling and subsequent implantation of tumor cells at the incision site.^(^^4^^)^

## CASE REPORT

A 53-year-old male patient, in outpatient follow-up with otolaryngology and clinical oncology teams at a university hospital, due to a laryngeal squamous cell carcinoma stage IV (T4N2M0 classification), treated with chemotherapy and radiation therapy, presenting with a complete response and evolving to cure. Upon diagnosis, tracheostomy and endoscopic gastrostomy were required.

**Figure 1:**
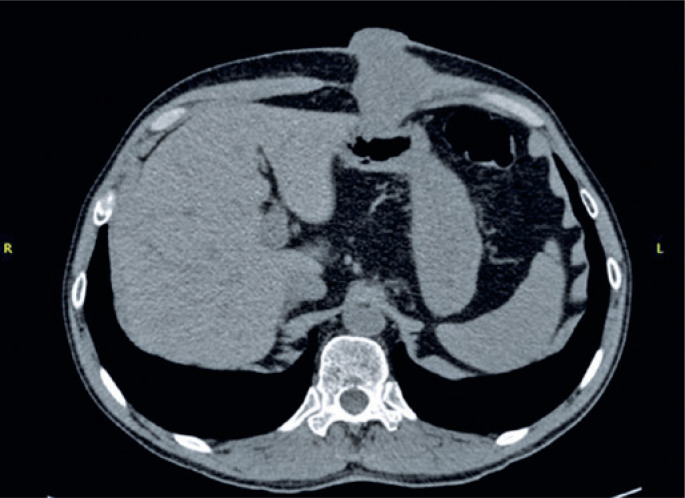
Non-contrast computed tomography, showing postoperative status of previous gastrostomy incision. Irregular contours with dense soft tissues adjacent to the surgical scar, with a component that externalizes, and is in intimate contact with the gastric wall, in the great curvature of gastric body, measuring approximately 56x55x52mm along its longest axes

The patient went to the general surgery outpatient clinic three months after remission of primary tumor, with an exophytic growth lesion in the gastrostomy orifice. He reported the onset of local pain and hyperemia two months before, evolving with hypertrophy of the skin, bleeding, subsequent skin necrosis, and fetid yellow secretion, which required several visits to the emergency department, where the gastrostomy probe was removed. Abdominal computed tomography was performed (Figure 1) and antibiotics were administered. The patient was referred to outpatient follow-up on several occasions due to worsening.

**Figure 2:**
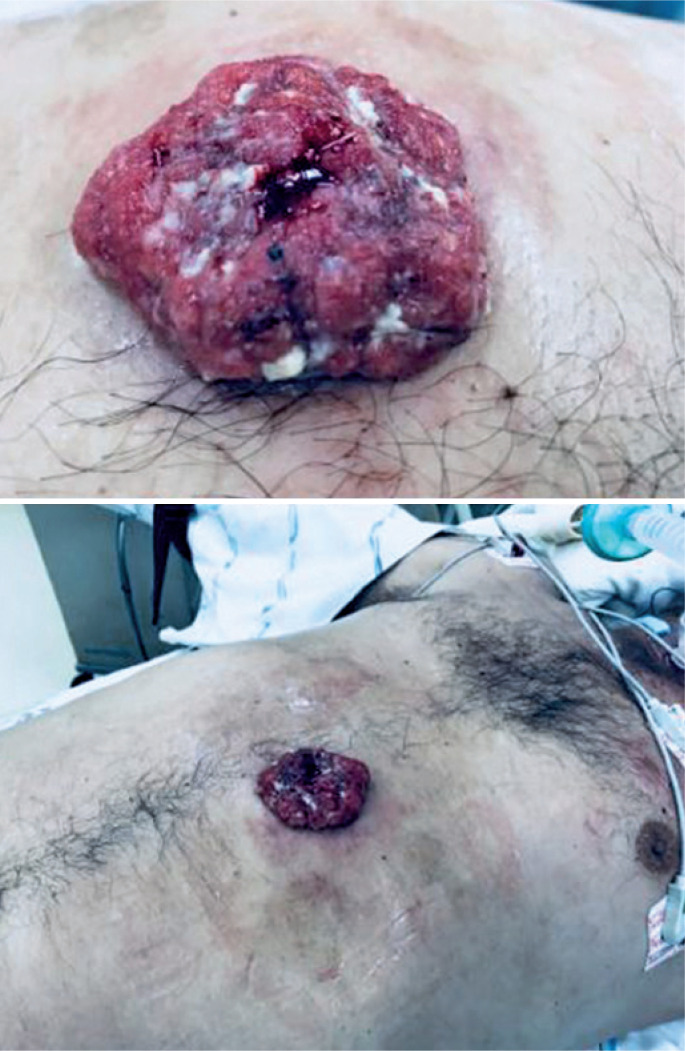
Extrusion of gastric mucosa through gastrostomy orifice

On physical examination during the outpatient visit, the patient presented with extrusion of gastric mucosa through the gastrostomy orifice (Figure 2), periostial hyperemia, skin hypertrophy measuring 0.5cm in diameter, granulation tissue, purulent and bloody discharge, and tenderness upon local palpation. The patient was admitted for an emergency procedure to correct gastric extrusion with exploratory laparotomy and gastrorrhaphy.

A median supraumbilical incision and opening by abdominal cavity planes were performed, and prolapse of the gastric mucosa through the gastrostomy orifice was observed. The gastric mucosa was detached from the abdominal wall, followed by a spindle incision in a previous gastrostomy area to excise the remaining mucosa adhered to the abdominal wall. Gastric suture was performed with simple continuous (running) and oversuture with 3-0 cylindrical mononylon thread, gastrostomy closure with Vicryl 1-0, aponeurosis closure with superlon 0, and skin closure with mononylon 3-0. The procedure was carried out with no complications, and the material excised was sent for pathological examination. The patient remained at hospital under monitoring for three days. He was discharged with instructions as to diet and return visit for subsequent outpatient follow-up.

The pathological report revealed a diagnosis of moderately differentiated and extensively invasive squamous cell carcinoma in the gastrostomy orifice, with involvement of fibrous scar tissue and subjacent fibrotendinous tissue. The (lateral peripheral) circumferential surgical margins showed involvement by the neoplasm, and the surgical margin in the (deep) adipose tissue presented no tumor.

## DISCUSSION

EPG has been the method of choice in patients with head and neck cancer (HNC),^(^^5^^)^ and metastasis by implantation of neoplastic cells is a rare complication. The probability of occurrence is believed to be less than one in one thousand cases,^(^^5^^)^ with a higher incidence in patients with advanced neoplasms.^(^^6^^)^

There are three hypotheses for this type of metastases at a gastrostomy site: due to translocation of tumor cells to the stoma, carried by the passage of the probe through the neoplasm; by hematogenic or lymphatic dissemination to the stoma, which supposedly is more susceptible because of prior surgical trauma and local vascularization conditions; or because of scaling tumor cells into the digestive tube, which later are implanted in the traumatized tissues of the stoma.^(^^2^^,^^6^^)^

There is evidence in literature that the stress generated by the surgical procedure can cause tumor metastasis, because of the high levels of cortisol after the intervention, which can induce morphological changes, both in the lumen of the capillaries and on the surface of the tumor, facilitating retention of neoplastic cells, which is compatible with the iatrogenic theory that direct implantation causes metastasis.^(^^3^^)^ Additionally, another theory is that damaged tissues are more susceptible to metastasis by hematogenic dissemination than are normal tissues, which would lead to implantation at the site of the incision.^(^^3^^)^ Cases in which the metastasis develops one year after the gastrostomy suggest hematogenic dissemination, due to the long interval; whereas rapid development suggests direct implantation of tumor cells.^(^^4^^)^

The theory of direct contamination is not yet clearly confirmed, but it is the only mechanism that physicians can avoid, by changing their approach. Evidence suggests that the method of choice for gastrostomy in patient with HNC should seek to avoid contact between the probe and the tumor tissue, since the passage of the probe can translocate tumor cells and implant them in the gastric mucosa.^(^^2^^)^ The technique to perform EPG should be individually chosen, considering case-by-case;^(^^7^^)^ however, the traction technique is preferred when there is no obstruction in the upper gastrointestinal tract, given the lower rate of short-term adverse events.^(^^6^^)^ Other options may be considered, such as the percutaneous technique with fluoroscope, since there is no need for a probe or its passage through the tumor site;^(^^2^^)^ performance of laparoscopy or open procedure (with the due care to maintain separate the surgical site and the equipment, in order to avoid contamination of the gastric mucosa, which might posteriorly generate new metastases); or even to consider chemotherapy or chemotherapy plus radiation therapy before EPG in an patient with the purpose of curing.^(^^6^^)^

The possibility of metastasis should always be taken into consideration in patients with HNC that present with skin changes at the gastrostomy site, which requires confirmation by biopsy.^(^^8^^)^ The risks of tumor implantation in the abdominal wall are higher in patients of advanced age and/or advanced tumor staging.^(^^6^^)^

## CONCLUSION

When the endoscopy gastrostomy probe is manipulated in the region of the oropharynx with neoplasia, there is risk of inducing metastases by direct implantation of neoplastic cells into the gastric incision. Despite this being a rare complication in endoscopic gastrostomy, we recommend that any contact between the probe and tumor tissue be avoided, if possible giving preference to the traction technique when there is no gastrointestinal obstruction, percutaneous technique with fluoroscope, laparoscope, or open procedure, and the choice of procedure should be made case-by-case by the professional in charge.

## References

[1] 1. Arends J, Bachmann P, Baracos V, Barthelemy N, Bertz H, Bozzetti F, et al. ESPEN guidelines on nutrition in cancer patients. Clin Nutr. 2017;36(1):11-48. 10.1016/j.clnu.2016.07.015 27637832

[2] 2. Laccourreye O, Chabardes E, Mérite-Drancy A, Carnot F, Renard P, Donnadieu S, et al. Implantation metastasis following percutaneous endoscopic gastrostomy. J Laryngol Otol. 1993;107(10):946-9. 10.1017/s0022215100124879 8263399

[3] 3. Sinclair JJ, Scolapio JS, Stark ME, Hinder RA. Metastasis of head and neck carcinoma to the site of percutaneous endoscopic gastrostomy: case report and literature review. JPEN J Parenter Enteral Nutr. 2001;25(5):282-5. 10.1177/0148607101025005282 11531220

[4] 4. Adelson RT, Ducic Y. Metastatic head and neck carcinoma to a percutaneous endoscopic gastrostomy site. Head Neck. 2005;27(4):339-43. Review. 10.1002/hed.20159 15712297

[5] 5. Rowell NP. Tumor implantation following percutaneous endoscopic gastrostomy insertion for head and neck and oesophageal cancer. Review of the literature. Head Neck. 2019;41(6):2007-15. 10.1002/hed.25652 30684284

[6] 6. Vincenzi F, De Caro G, Gaiani F, Fornaroli F, Minelli R, Leandro G, et al. Risk of tumor implantation in percutaneous endoscopic gastrostomy in the upper aerodigestive tumors. Acta Biomed. 2018;89(8-S):117-21. 10.23750/abm.v89i8-S.7894 PMC6502208 30561429

[7] 7. Retes FA, Kawaguti FS, Lima MS, Martins C, Uemura RS, Paulo GA, et al. Comparison of the pull and introducer percutaneous endoscopic gastrostomy techniques in patients with head and neck cancer. United European Gastroenterol J. 2017;5(3):365-73. 10.1177/2050640616662160 PMC5415208 28507748

[8] 8. Greaves JR. Head and neck cancer tumor seeding at the percutaneous endoscopic gastrostomy site. Nutr Clin Pract. 2018;33(1):73-80. Review. 10.1002/ncp.10021 29323421

