# Supplementary material for: Squamous cell carcinoma implantation in gastrostomy orifice. Case report
Source: Einstein (Sao Paulo). 2020 Oct 29;18:eRC5409. doi: 10.31744/einstein_journal/2020RC5409 (PMC7607919; doi:10.31744/einstein_journal/2020RC5409)
Supplement: Translation

# Translation: Implantação de carcinoma espinocelular em orifício de gastrostomia. Relato de caso

**Authors:** Ana Flávia Azevedo Querichelli, Fernanda Luiza Faria, Laura Ferreira Martinez, Eumildo de Campos

**Affiliations:** 1 São José do Rio PretoSP Brasil Faculdade de Medicina de São José do Rio Preto, São José do Rio Preto, SP, Brasil.

**Keywords:** Gastrostomia, Carcinoma de células escamosas, Metástase neoplásica, Neoplasias de cabeça e pescoço, Nutrição enteral

## Abstract

A gastrostomia endoscópica percutânea é utilizada para oferecer suporte nutricional enteral para pacientes com neoplasias obstrutivas de orofaringe ou esôfago. A colocação da sonda é considerada segura, com poucas complicações. Apesar disso, uma complicação em particular, considerada rara, está sendo cada vez mais descrita na literatura: a metástase de neoplasia de cabeça e pescoço para o estoma da gastrostomia. Neste relato, descrevemos um caso de metástase de carcinoma espinocelular de laringe para o sítio da gastrostomia, e discutimos as possíveis etiologias e alternativas, buscando diminuir a incidência desta complicação.

## INTRODUÇÃO

A gastrostomia endoscópica percutânea (GEP) é utilizada como via alternativa de alimentação entérica, quando a via oral está prejudicada, e o paciente não é capaz de nutrir-se adequadamente, buscando evitar desnutrição e caquexia.^(^^1^^)^ É uma alternativa à gastrostomia cirúrgica ou sonda nasogástrica em pacientes que necessitam de suporte nutricional a longo prazo.^(^^1^^)^ Está indicada em casos de neoplasias obstrutivas de laringe, faringe ou esôfago, dificuldades na deglutição por doença neurológica ou radioterapia, e trauma facial.^(^^2^^)^

Quando a sonda utilizada para realização da gastrostomia endoscópica é manipulada na região da orofaringe com neoplasia, esta pode entrar em contato com o tumor, podendo induzir metástases por implantação direta de células neoplásicas em sítio aberto por incisão na superfície gástrica pela passagem da sonda,^(^^3^^)^ ou devido à descamação e à posterior implantação de células tumorais no local da incisão.^(^^4^^)^

## RELATO DE CASO

Paciente do sexo masculino, de 53 anos, em acompanhamento ambulatorial com as equipes da otorrinolaringologia e oncologia clínica em um hospital universitário, devido a carcinoma espinocelular em laringe estágio IV (classificação T4N2M0), que foi tratado com sessões de quimio e radioterapia, tendo resposta completa e evoluindo para cura. Na época do diagnóstico, foram necessárias traqueostomia e gastrostomia endoscópica.

Paciente compareceu ao ambulatório da equipe da cirurgia geral após três meses da remissão tumoral primária, com lesão extrusiva de crescente volume em orifício da gastrostomia. Referia que 2 meses antes iniciou quadro de dor e hiperemia locais, evoluindo com hipertrofia de pele, sangramento, posterior necrose de pele e secreção amarelada fétida, sendo necessárias diversas consultas no serviço de emergência, onde foi retirada a sonda de gastrostomia, tendo sido realizadas tomografia computadorizada abdominal (Figura 1) e antibioticoterapia, além de encaminhamento ambulatorial, em diferentes momentos, devido à piora apresentada.

**Figura 1:** Tomografia computadorizada sem contraste, evidenciando status pós-operatório de rafia de gastrostomia prévia. Contornos irregulares com densidade de partes moles adjacente à cicatriz cirúrgica, com componente que se exterioriza e também em íntimo contato com a parede gástrica na topografia do corpo gástrico na grande curvatura, medindo, aproximadamente, 56x55x52mm em seus maiores eixos

Ao exame físico da consulta ambulatorial, paciente apresentava extrusão da mucosa gástrica por meio de orifício da gastrostomia (Figura 2), hiperemia periostial, hipertrofia de pele de 0,5cm de diâmetro, tecido de granulação, secreção purulenta e sanguinolenta, e dor à palpação local. Diante do quadro, foi optado pela internação do paciente, para realização de procedimento de urgência, para correção da extrusão gástrica, com laparotomia exploradora e gastrorrafia.

**Figura 2:** Extrusão de mucosa gástrica através de orifício de gastrostomia

Foram realizadas incisão mediana supraumbilical e abertura por planos da cavidade abdominal, sendo verificado prolapso de mucosa gástrica pelo orifício da gastrostomia. Realizou-se descolamento entre mucosa gástrica e parede abdominal, seguida de incisão em fuso em área de gastrostomia prévia, para excisão de mucosa remanescente aderida à parede abdominal.

Foi realizada rafia gástrica com chuleio simples e sobressutura com fio mononylon cilíndrico 3-0, fechamento do orifício da gastrostomia com Vicryl 1-0, fechamento da aponeurose com superlon 0 e fechamento da pele com mononylon 3-0. O procedimento foi realizado sem intercorrências, e o material extraído foi enviado para análise anatomopatológica. O paciente permaneceu internado e monitorado por 3 dias, recebendo alta com orientação sobre dieta e retorno para acompanhamento ambulatorial posterior.

O relatório do exame anatomopatológico evidenciou diagnóstico de carcinoma espinocelular moderadamente diferenciado e extensamente invasivo em orifício de gastrostomia, com envolvimento de tecido fibroso cicatricial e tecido fibrotendíneo subjacente. As margens cirúrgicas circunferenciais (periférico lateral) revelaram comprometimento pela neoplasia, e a margem cirúrgica no tecido adiposo (profundo) estava livre de neoplasia.

## DISCUSSÃO

A GEP tem sido o método de escolha em pacientes com câncer de cabeça e pescoço (CCP),^(^^5^^)^ e a metástase por implantação de células neoplásicas é uma complicação rara, acreditando-se que a probabilidade de ocorrência seja menor que um em mil casos,^(^^5^^)^ sendo mais incidente em pacientes com neoplasias avançadas.^(^^6^^)^

Há três hipóteses para esse tipo de metastização em sítio de gastrostomia: devido à translocação de células tumorais para o estoma, arrastadas pela passagem da sonda por meio da neoplasia; pela disseminação por via hematogênica ou linfática até o estoma, que supostamente seria mais suscetível, devido ao trauma cirúrgico prévio e a condições locais de vascularização; ou devido à descamação de células tumorais para o tubo digestivo, que posteriormente são implantados nos tecidos traumatizados do estoma.^(^^2^^,^^6^^)^

Há evidências na literatura de que o estresse gerado pelo procedimento cirúrgico pode causar aumento na metástase tumoral, devido aos altos níveis de cortisol após a intervenção, que podem induzir alterações morfológicas, tanto no lúmen dos capilares quando na superfície tumoral, facilitando retenção de células neoplásicas, o que é compatível com a teoria iatrogênica de que a implantação direta causa metástase.^(^^3^^)^ Além disso, outra teoria é que tecidos danificados sejam mais suscetíveis a levar a metástases por disseminação hematogênica do que tecidos normais, levando, então, à implantação no sítio da incisão.^(^^3^^)^ Casos em que a metástase se desenvolve após 1 ano da realização da gastrostomia sugerem disseminação hematogênica, devido ao longo intervalo; enquanto o desenvolvimento rápido sugere implantação direta das células tumorais.^(^^4^^)^

Embora a teoria da contaminação direta não esteja claramente comprovada, este é o único mecanismo que pode ser evitado pelo médico por meio de mudanças em sua abordagem. Evidências sugerem que o método de escolha para realização de gastrostomia em paciente com CCP deve buscar evitar o contato entre a sonda e o tecido tumoral, uma vez que a passagem da sonda pode translocar células tumorais e implantá-las na mucosa gástrica.^(^^2^^)^ A técnica de escolha para realização da GEP deve ser feita individualmente, considerando caso a caso,^(^^7^^)^ no entanto a técnica de tração é preferida quando não há obstrução do trato gastrintestinal superior, dada a menor taxa de eventos adversos a curto prazo.^(^^6^^)^ Outras opções podem ser consideradas, como a técnica percutânea com fluoroscópio, uma vez que não necessita de sonda e nem da passagem desta pelo sítio tumoral;^(^^2^^)^ a realização de laparoscopia ou procedimento aberto para posicionamento da sonda (com os devidos cuidados para manter o sítio cirúrgico e o equipamento separados, buscando evitar contaminação da mucosa gástrica, que possa posteriormente gerar novas metástases); ou até mesmo considerar a quimioterapia ou quimioradioterapia antes da GEP em paciente com intenção de cura.^(^^6^^)^

A possibilidade de metástase deve sempre ser considerada em pacientes com CCP que apresentem alterações dermatológicas no sítio da gastrostomia, necessitando ser confirmadas com biópsia.^(^^8^^)^ Os riscos de implantação tumoral na parede abdominal são maiores em pacientes de idade avançada e/ou estadiamento tumoral avançado.^(^^6^^)^

## CONCLUSÃO

Quando a sonda da gastrostomia endoscópica é manipulada na região da orofaringe com neoplasia, há risco de indução de metástase por implantação direta de células neoplásicas na incisão gástrica. Apesar de esta ser uma complicação rara da gastrostomia endoscópica, recomenda-se evitar o contato entre sonda e tecido tumoral, dando preferência à técnica de tração quando não há obstrução gastrintestinal, técnica percutânea com fluoroscópio, laparoscopia ou procedimento aberto, devendo a escolha do procedimento ser feita caso a caso pelo profissional responsável.
